# Public engagement with science—Origins, motives and impact in academic literature and science policy

**DOI:** 10.1371/journal.pone.0254201

**Published:** 2021-07-07

**Authors:** Peter Weingart, Marina Joubert, Karien Connoway

**Affiliations:** 1 Centre for Research on Evaluation, Science and Technology (CREST), Stellenbosch University, Stellenbosch, South Africa; 2 Institute for Interdisciplinary Studies of Science, Bielefeld University, Bielefeld, Germany; 3 South African Research Chair in Science Communication, Centre for Research on Evaluation, Science and Technology (CREST), Stellenbosch University, Stellenbosch, South Africa; Universita degli Studi di Foggia, ITALY

## Abstract

‘Public engagement with science’ has become a ‘buzzword’ reflecting a concern about the widening gap between science and society and efforts to bridge this gap. This study is a comprehensive analysis of the development of the ‘engagement’ rhetoric in the pertinent academic literature on science communication and in science policy documents. By way of a content analysis of articles published in three leading science communication journals and a selection of science policy documents from the United Kingdom (UK), the United States of America (USA), the European Union (EU), and South Africa (SA), the variety of motives underlying this rhetoric, as well as the impact it has on science policies, are analyzed. The analysis of the science communication journals reveals an increasingly vague and inclusive definition of ‘engagement’ as well as of the ‘public’ being addressed, and a diverse range of motives driving the rhetoric. Similar observations can be made about the science policy documents. This study corroborates an earlier diagnosis that rhetoric is running ahead of practice and suggests that communication and engagement with clearly defined stakeholder groups about specific problems and the pertinent scientific knowledge will be a more successful manner of ‘engagement’.

## 1. Introduction

The ‘engagement discourse’ has assumed a prominent role in the science policy rhetoric in the European Union (EU), the United States of America (USA) and beyond, to medium-income countries such as South Africa (SA). Bensaude-Vincent diagnosed the engagement rhetoric as ‘buzz’, having its origin in management and marketing [1, p. 239]. ‘Buzz’, in this context, stands for the polysemy of a particular concept that allows for a multitude of usages and its capacity to make sense in different discourses [2, p. 20].

Concepts, metaphors or more generally rhetoric, serve a multitude of actors and their interests. They mediate between different institutions, may legitimate or de-legitimate positions of power, and signal societal change [2]. The ‘science engagement’ rhetoric is no exception. Its ‘success’, i.e. its spread in academic literature and in science policy documents is puzzling, at least on the surface, as it challenges several institutional arrangements: the sources of democratic legitimacy, the functional differentiation between politics and science, and cultural and socio-economic differences among nations.

Firstly, the term ‘engagement’ is side-lining the conflict characterizing modern democracies, between political representatives, legitimated by popular vote, and experts legitimated by expertise based on scientific knowledge, or between democratic and technocratic rule. The conflict between these two sources of legitimacy has increased in intensity for two main reasons. On the one hand, problems of government have become more complex and thereby the need to rely on scientific knowledge represented by experts has grown, conferring more actual power on them. On the other hand, citizens’ rights and claims to political influence have expanded over the past two centuries [3].

Secondly, ‘public engagement’ ignores the functional differentiation of social systems, especially between the roles of citizens and of professionals. Participation in political debates and decision-making in a democracy is open to every citizen, regardless of qualification. Science, conversely, is a highly specialized social system participation in which requires, in most cases, the completion of years of advanced higher education with some kind of certification as the entry ticket to a professional role. Participation, in the sense of engagement, normally does not intend the acquisition of professional expertise. Nevertheless, forms and degrees of participation in science by non-scientists vary considerably, e.g., depending on the accessibility of the disciplines concerned and the kind of contributions expected from citizens. These range from data collection in large spatial and temporal scales (e.g., in biology, environmental studies and optical astronomy) to actual innovation through the exchange of information [4, 5] and ultimately to generating scientific knowledge [6, 7]. Macq, Tancoigne and Strasser [8] distinguish between “participation in decision-making” (pertaining to science policy decisions or decisions on research topics) and “participation in knowledge and innovation-making,” for their discourse analysis. Thus, the ‘public engagement’ rhetoric reflects different forms and degrees of participation of citizens in governments’ decisions and the provision of expert knowledge [9, p. 217]. The diverging concepts of ‘public engagement’ in essence, represent two different models of direct and representative democracy. The call for ‘public engagement with science’ mainly from the science and technology studies (STS) community, with the normative expectation of a ‘democratization’ of science, reflects its members’ critical stance towards representative or ‘elite’ democracy and their attraction to concepts of direct democracy carried over from the social movements of the 1960s and 1970s [10, p. 591]. This call has subsequently been taken up by science policymakers, thus becoming an initiative ‘from above’ attempting to nudge ‘the public’ into engaging with science [11]. Skeptical observers have drawn attention to the paradox of the engagement rhetoric itself: the engagement that is supposed to be a dialogue at eye level between scientists and the public is nevertheless initiated and orchestrated by scientists, their organizations, or governments [1, p. 244, 12, p. 43, 13]. This suggests that the very term ‘engagement’ will assume many different meanings, depending on who propagates it and initiates its implementation.

Finally, a further aspect of the ‘success’ of the rhetoric is its global spread, though with some variation, across the socio-economic and cultural differences and differences in political and science systems between nations. Neo-institutionalists have identified this phenomenon as isomorphism by imitation [14]. In fact, it is highly likely that scholars and science policy actors worldwide follow each other, copying the engagement rhetoric, whatever their particular motives are, simply because it is ‘the thing to propagate’.

In this study, we trace the origins and evolution of the discourse on ‘public engagement with science’, both in the academic discourse and in the broader political context. We aim to identify the different motivations for engagement and how they are undergoing change in the transition of the concept from one context to the other. The ultimate question is whether it is likely to succeed in bridging the gap between the popular representative and the expert knowledge-based types of legitimacy, i.e. in mediating between representative democratic and technocratic forms of governance.

## 2. Selective literature review

Two recent studies are especially pertinent to our own. They focus on the emergence of the discourse on ‘public engagement’, the conceptual vagueness of the term itself, the motives behind it, i.e. the functions that are associated with it, and the obstacles that frustrate its implementation [8, 15]. These studies ask similar questions and cover partly the same ground; only their scopes of analysis and methodologies differ from our study. Conceição et al. analyze the last four European science policy framework programs (FP5-7, H2020) and ask, “how much terminologies, meanings, and foci of attention have changed” [15, p. 1]. They conclude that there is a “relative devaluation of initiatives oriented towards the fields of science education, the public communication of science and the promotion of a scientific culture” [15, p. 20]. Macq, Tancoigne and Strasser also focus on the science policy of the EU and look at institutional dynamics and epistemic communities as the factors “shaping the policies of participation”, to shed light on how the “conception and promotion of public participation in European science and technology policy evolved over time” [8, p. 489]. They find that “while public participation had initially been conceived and promoted as a way to build legitimacy of research policy decisions by involving publics into decision-making processes, it is now also promoted as a way to produce better or more knowledge and innovation by involving publics into knowledge and innovation-making processes, and thus building legitimacy for science and technology as a whole” [8, p. 508].

One issue discussed with respect to participation and engagement is the different forms they may take. The dichotomous distinction between ‘invited’ and ‘uninvited’ participation or engagement is fundamental in this context [11, p. 107, 16]. Uninvited participation is unpredictable and as such, exactly what governments and vested interest groups try to avoid and pre-empt. However, by addressing, i.e. ‘inviting’ publics to engage with science, the “normative social commitments projected and performed by science” are already accepted [11, p. 108, 12, p. 43]. According to this view, expressed primarily by scholars in the STS community, invited engagement, albeit at least a step in the direction of greater transparency and accountability, is not sufficient.

The typology of governance suggested by Hagendijk and Irwin [17, p. 172] provides a more differentiated and realistic picture of the variations of the actors’ roles in initiating and executing science policy decisions. It illustrates that the supposed empowerment of citizens, as demanded by early proponents of engagement, cannot and does not escape the political context in which the terms of discussion and prior commitments to values and material conditions, have already been defined. This is amply apparent in the dilemma facing policymaking between securing legitimacy by ‘public engagement’ on one hand and allowing for innovation by unrestrained science on the other, as stated in the House of Lords Third Report [18].

Rowe and Frewer [19, p. 254] base their definition of engagement on the flow of information and thus differentiate between public communication, public consultation, and public participation. Only in the latter is the flow of information bi-directional between ‘sponsor’ and public representatives. They list more than 100 ‘mechanisms’ that may be subjected to this definition, leading them to admit to an “uncertain and contradictory nomenclature” that creates “confusion and term proliferation” [19, pp. 258–259]. As the discourse appears to have shifted from participation and deliberation to ‘engagement’, the level of democratization in terms of the public’s involvement in science policy decisions and research proper, has been raised successively in the evolution of the discourse. This is amply illustrated by the plethora of meanings associated with the term ‘engagement’, as well as the multitude of mechanisms listed under the label [19].

The diverse forms of engagement respond to two threats of legitimacy: 1) the threatened legitimacy of science (represented by experts and official spokespersons) and 2) the threatened legitimacy of governments. Both threats manifested in public protests against particular decisions about scientific and technological programs. These threats have increased in urgency since science has become a crucial factor in national economies, thus increasing their competition for innovation advantages. ‘Engagement’ is promoted with the expectation that involving the public in science will contribute to knowledge production and the improvement of the workforce, thereby augmenting innovative capacity [20]. All these motives point to an overarching objective to secure the public’s acceptance of scientific and technological developments.

The issue of legitimation as an underlying motive of the ‘engagement’ rhetoric raises the question of the kinds of publics it addresses. When, in the 1970s, student activists at universities set up ‘science shops’, the precursors of present citizen science projects, they addressed the local civil society and NGOs as their clientele [21]. When the technology assessment movement came of age, randomly polled citizens, (‘micro publics’) were usually selected to participate in consensus conferences, citizen juries and similar arrangements. A local or regional reference of the issue to be dealt with, as e.g., in the case of municipal budgeting, often served as a criterion for selection [22, 23].

As the rhetoric became more ambitious and shifted to ‘public engagement with science’, it also became less specific with respect to who this public is that is supposed to be engaged with or be engaged by science [24]. The implicit definition of the ‘public’ avoids the issues of democratic representation, not only because of its un-specificity, but also because it does not stipulate any particular action. The ‘public’ means different groups to different actors, and both governments and private (science-based) industry have different interests, such as securing legitimacy or protecting investments, than scholars propagating ‘engaging’ the public in deliberations about science and technology policy issues or the risks and benefits of new technologies [25].

This departure from earlier, more focused forms of participation is reflected in the increasing vagueness of programmatic language in policy documents. In 2008, the UK Department for Innovation, Universities and Skills (DIUS) stated: “We are using ‘public engagement’ to be an *umbrella term*–that encompasses many kinds of activity including science festivals, centers, museums, cafes, media, consultations, feedback techniques, and public dialogue” [26, p. 19, our italics]. In 2019, the National Coordinating Centre for Public Engagement (NCCPE), set up in the meantime, was even vaguer: "Public engagement describes the *myriad of ways* in which the activity and benefits of higher education and research can be shared with the public” [27, our italics].

It is just this overburdening of the term with good intentions to vitalize democracy, proliferation of organizational formats of engagement and intensive expectations in their functioning, that led Hagendijk and Irwin to state that “rhetoric is running well ahead of practice” because the reality on the ground lags far behind [17, p. 176].

Since this critical diagnosis of the dynamics of the ‘engagement’ rhetoric, some skepticism seems to have spread more recently in the academic community. Conceição et al. [15, p. 6] note: “many academics have acknowledged that the expectations for new models of communication and participation in the governance of science are far from completely fulfilled”. They point to the “practical difficulties involved in implementing the proposed new models, the latent ambiguity (or even contradictions) in their objectives, their frequent inconsequence in practical terms, or even the perverse effects generated by the implementation of such ideas” [15, p. 6, 28].

This divergence of the rhetoric of legitimation and its implementation was already an issue in the political science discourse when it took the ‘participatory turn’ in the 1960s [29, p. 225]. Ganuza et al. distinguish the participation movement in the 1960s, that was emancipatory and directed against ruling political and economic elites, from the present in which it is “understood as complementary to the social order” [22, p. 329]. The various experiments with consensus conferences, planning cells, roundtables, citizen juries, etc., actually responded to broader concerns about a growing distance between experts and the citizenry or, to put it differently, to an increasing gap of legitimacy for democratic governments. In order to bring citizens ‘back’ into politics and curb the influence of technocratic expertise, participation models involving citizens were put into practice [22].

However, the high expectations in the legitimating function of participatory formats that inevitably involved small ‘publics’ have been sobered by theoretical considerations regarding the relation of these formats to representative democracy. Subsequently, the discourse shifted from the participation of citizens’ groups to ‘deliberation’ as a source of democratic legitimacy [30, 31]. In deliberative models, legitimacy is created by a rational deliberation among citizens [32, 33]. It is not just *who* makes decisions, but *how* they are made, i.e. the *quality* of decisions that is the issue [32, p. 191]. One of the many meanings of ‘engagement’ is a dialogue between scientists and the public, which allows for *mutual learning*. This appears as an extension of the participation and deliberation paradigms. While, in theory, a combination of citizens’ participation in decision-making and deliberation would enhance democratic legitimacy, in practice there are numerous obstacles and contradictions [32, 34–36]. The rhetorical shift from participation to ‘engagement’ reflects this step from participation to deliberation. ‘Engagement’ likewise lost specificity of meaning compared to ‘participation’.

In the narrower area of science communication, the development of this discourse was mirrored by the shift from ‘public understanding of science’ to ‘public engagement with science’. The academically dominated argument started with concerns about science literacy among the general public in the 1960s [37, p. 80–85]. In the logic of the ‘empowerment’ of the citizenry, this soon came to be criticized as implying a ‘deficit’ of knowledge about science, as defined by what the scientific elites determined to be important to know. From 1985 onwards, this was followed by the ‘public understanding of science’ paradigm which also assumed a deficit of understanding, but focused on a change of attitudes among the public, with the consequence that it motivated public relations (PR) campaigns instead of the previous education-oriented programs. However, the expectation, that better knowledge of science would generate acceptance, proved wrong. From the 1990s onwards, the solution to rebuild trust in science was seen in public participation and deliberation, though to varying degrees in the EU and the USA.

In order to contextualize the engagement discourse, it has to be recalled that its origin lies in the discussions about technology assessment and the credibility crisis of scientific expertise. The critique of scientific elites held responsible for new technologies, notably nuclear energy, first surfaced in the public discourse in the USA in 1969 with the foundation of the Union of Concerned Scientists. The 1970s saw the Energy Reorganization Act with a new regulatory régime for nuclear energy (1974), and the accidents at the Browns Ferry (March 1975) and Three Mile Island nuclear power plants (March 1979). In Europe, more so than in the USA, the accident at the Chernobyl reactor in 1986 alongside the bovine spongiform encephalopathy (BSE) crisis (1989–1996) and the protracted debate over genetically modified (GM) food of 1999 and beyond, all contributed to the erosion of public trust in science and scientists. In the USA, a similar effect was caused by debates about climate change, reproductive technologies, and cloning [1]. The repetition and growing intensity of public protests facing the, at the time still prevalent, style of top-down and at times delusive, communication both from policymakers as well as their scientific advisers, became a threat to the general trust in science and the legitimacy of governments.

The logic of technology assessment implied a gradual widening of public participation in political decision-making about scientific and technological issues. At first, the public was to be involved in risk assessment, but it soon became evident that, once a new technology was established, it was too late to change it, let alone reverse its implementation. This called for moving technology assessment ‘upstream’, i.e. involving the public, in principle, already at the stage of the conception of new technologies [38]. An institutional innovation that attempted to meet this challenge was the ‘consensus conference’, first set up by the Danish Board of Technology in the 1980s [39]. The Danish model was to involve ‘ordinary citizens,’ after they were introduced by experts to the respective technicalities, in techno-scientific decisions. This was considered a ‘democratization’ of science policy decision-making [40].

Since the publication of the Bodmer Report in 1985, strong evidence suggests that the academic community, represented by STS scholars on one side and policymaking bodies, first in the UK, then in other European countries and the USA on the other, had different motives for propagating public engagement with science. While the STS community led an intellectual discourse directed at the ‘democratization of science’, the political side, including public science organizations like research councils, academies, professional societies and universities, tried to cope with threats to legitimacy–the legitimacy of governments fearing public protests and the legitimacy of science both as an institution and as a crucial factor in economic and innovation policy. On the surface, at least, both sets of motives converge on the call for ‘public engagement with science’ [41].

**Fig 1:**
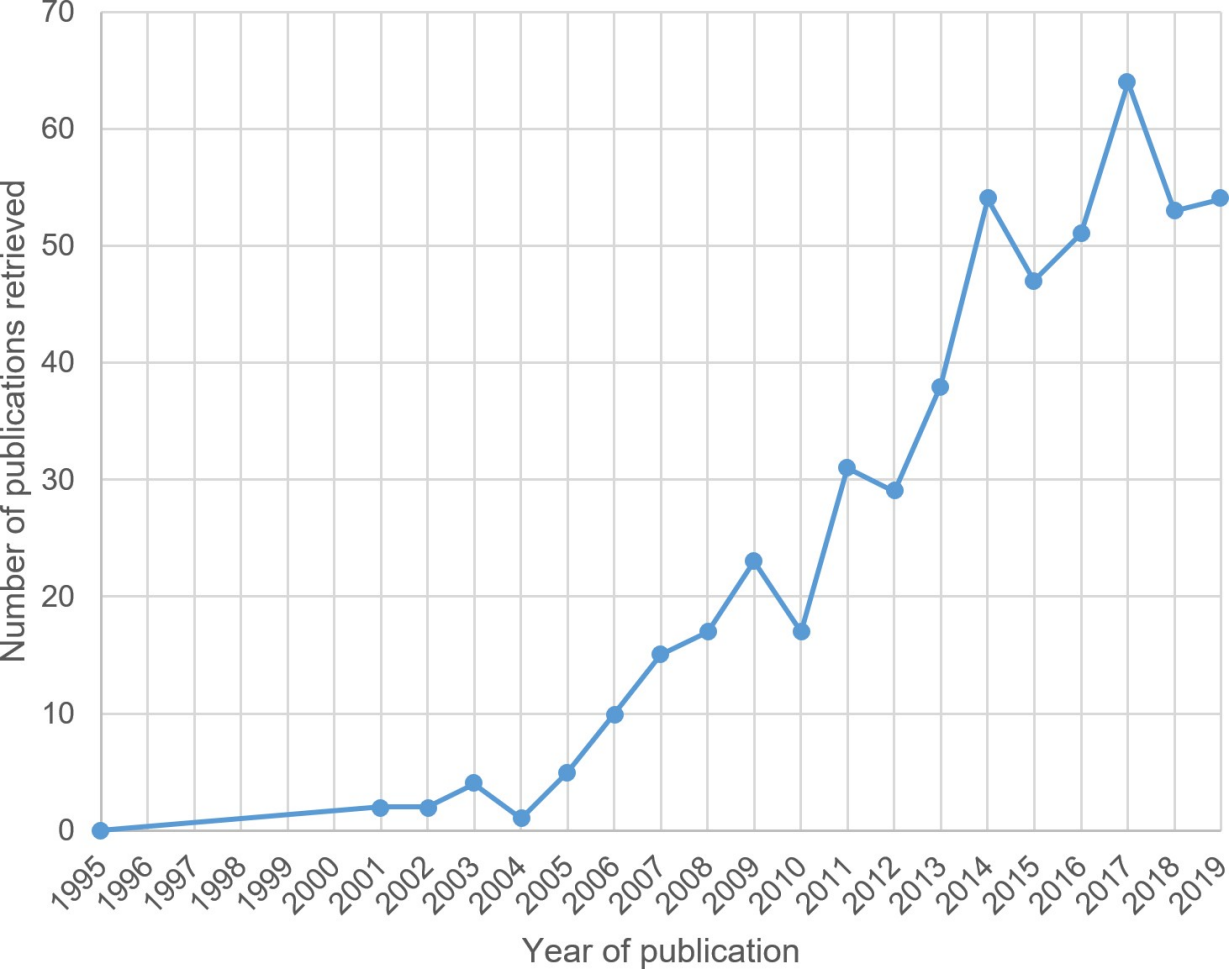
Growth in the occurrence of the combination of the terms ‘public engagement’ and ‘science’ in titles or abstracts in the Web of Science from 1995–2019 (N = 517).

According to Hagendijk and Irwin, “since the late 1990s, talk of engaging ‘the public’ in scientific and technological change has become fashionable in policy circles, and especially in Europe” [17, p. 167]. The ‘engagement’ rhetoric really took off in the early 2000s (see Fig 1). Since then, the meanings of ‘engagement’ have evolved further. One possible meaning is focused on socially relevant knowledge production, or, seen from the political side, the demonstration of impact. In this context, ‘citizen science’ and ‘community-based participatory research’, and the related concepts of ‘anticipatory governance’ and ‘responsible innovation’ are pertinent [42, p. 21]. The latter appears to have gained dominance in the science policy discourse, thus prioritizing the instrumental role of science for innovation and economic return [43–46].

Given the prominence of the ‘engagement rhetoric’ in academic discussions and in science policies, we investigate the rhetoric in detail to determine its substance as well as its likelihood of overcoming the gap between science and society. In the following empirical analysis we trace the academic discourse on engagement in three journals devoted specifically to science communication, as well as the main science policy programs on ‘engagement’ in the UK, USA, EU and SA. Our content analysis is guided by the following research questions (RQs):

RQ1: How is ‘public engagement with science’ defined in the academic literature and in the policy documents?
RQ2: What motivations are attributed to ‘public engagement with science’ in the academic literature and in the policy documents?
RQ3: What are the main criticisms levelled against ‘public engagement with science’ in the academic literature and in the policy documents, especially with regard to its implementation?
RQ4: What is the relation between the academic and the political discourses on ‘public engagement with science’?

## 3. Methodology

In order to obtain an overview of the presence of ‘public engagement’ in connection with ‘science’ we conducted a bibliometric analysis based on Web of Science (WoS) data. This provided the base for selecting a sample of articles for a content analysis aimed at understanding the development of the ‘engagement’ rhetoric. Furthermore, this was intended to return insights regarding dominant journals and country affiliations in the discourse around ‘public engagement’ and ‘science’.

To gain a better understanding of how the term ‘engagement’ is conceptualized in academic articles and policy documents, we created and analyzed samples of 86 academic articles and 19 policy documents, using a process of systematic content analysis as described by Bryman [47, pp. 295–299]. Specifically, we employed thematic coding to answer key questions related to ‘engagement’ and its definitions, motivations and criticisms. Systematic content analysis using thematic coding is particularly appropriate for our data since it allows for longitudinal analysis and has the necessary flexibility to enable us to use the same method for both the academic articles and policy documents [47, p. 304].

### 3.1. Sampling and analyzing academic articles focused on ‘engagement’

**Fig 2:**
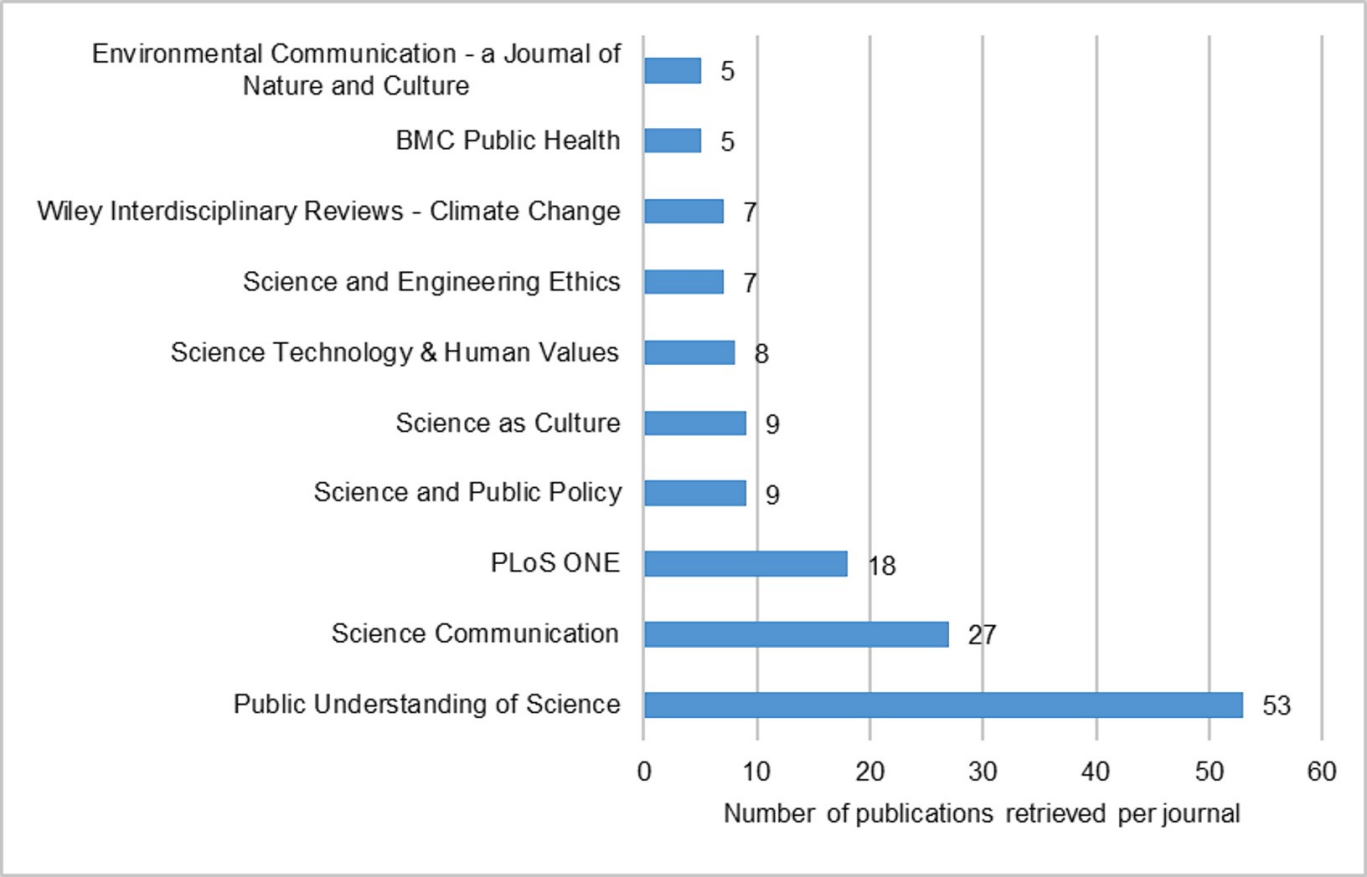
Top 10 academic journals among publications with a combination of the terms ‘public engagement’ and ‘science’ in their titles or abstracts in the Web of Science for the period 1995–2019.

The systematic procedure used to identify the sample of academic articles for coding is presented in Table 1 below. Since *Public Understanding of Science* (*PUS*) and *Science Communication* (*SC*) were identified as the academic journals in which the combination of ‘public engagement’ and ‘science’ features most prominently (See Fig 2 below) they were considered credible sources to provide insight into how public engagement with science is understood and debated in the academic community. Additionally, we included the *Journal of Science Communication* (*JCOM*). *JCOM* is currently listed only in the Emerging Sources Citation Index of the WoS. (This indicates that *JCOM* is in a probationary stage from which it may eventually enter more advanced indexes such as the Social Sciences Citation Index). Nonetheless, it is already a prominent journal in the field of science communication [48]. Moreover, it is the only open-access journal devoted to the field.

**Table 1:** Search and selection process of academic articles.

Steps in search and selection process of academic articles	Number of articles
Searched for articles with the terms ‘public engagement’ and ‘science’ in either titles or abstracts in the Web of Science	517
	Main journals: *PUS* and *SC*
Searched for the keyword ‘engag*’ (which would capture the terms engage, engaged, engaging and engagement) in the online databases of *JCOM*, *PUS* and *SC* (from date of first publication for each journal until the end of 2018)	455
Selected occurrences of engagement in titles and/or abstracts and the context of science communication in *JCOM*, *PUS* and *SC*	284
	*JCOM*: 78
	*PUS*: 130
	*SC*: 76
Applied a multi-stage, stratified sampling method proportionate to year and journal with a threshold criterion of five articles per year. Where the number of articles per year exceeded five, purposeful selection (according to title and abstract) was carried out proportionate to the number of articles per journal.	86

The final sample of 86 articles was coded thematically guided by a codebook consisting of 18 (eight descriptive and ten interpretive) variables. Each of the interpretive variables required the coder to answer a specific set of questions. The key objective of the codebook was to determine how ‘engagement’ is defined, as well as to identify key motivations and criticisms relating to engagement. We tested our codebook on 10% of the sample to ascertain whether the questions were formulated clearly and with relevance to our research questions. (See S1 Appendix for the complete codebook). Inter-coder reliability was calculated according to Cohen’s Kappa (*k*) after two coders coded the same 10 articles (12% of the sample). The following satisfactory results were obtained: descriptive variables (*k* = .88), interpretive variables (*k* = .83).

### 3.2. Sampling and analyzing policy documents focused on ‘engagement’

In terms of the policy discussion, we chose to focus on four different regions, namely the United Kingdom (UK), United States of America (USA), and the European Union (EU), i.e. those most prominent in science and technology, in addition to South Africa (SA) as a middle-income country. Based on the researchers’ knowledge of the organizations responsible for science policymaking in these four regions, we searched for policy directives issued by organizations responsible for science policymaking. We began by looking for policies issued at the level of government departments, such as white papers and government strategies. We included documents containing directives for researchers about the mandatory or optional integration of public engagement into their research activities. In the case of the USA, there is no single body or agency responsible for policymaking regarding science engagement [49]. Therefore, we opted to include relevant policy directives from a major funding agency (The National Science Foundation) and a broad-based science academy (The National Academies of Sciences Engineering and Medicine), based on the close proximity of these institutions to political decision-making. It is worth noting that, in 2020, the ‘Day One Project’ proposed a new federal strategy for science engagement [50]. At the level of the EU, we included policies issued by EU-wide research collaboratives, such as the EU Horizon 2020 Programme. Since we were specifically interested in exploring science policy with relevance to public engagement with science, our core selection criteria for inclusion of policy documents was that it should make direct reference to the term “engagement”. As such, earlier policy documents referring to terms such as ‘science literacy’ or ‘public understanding of science’ were not included.

Based on this search strategy and selection criteria, we selected 19 policy documents for further analysis. Although some descriptive codes in the codebook naturally had to be adapted for the policy context (see S1 Appendix for details) all interpretive codes remained the same. Again, the codebook was tested with 10% of the sample and inter-coder reliability was calculated according to Cohen’s Kappa (*k*) after two coders coded the same three policy documents (15% of the sample). The following satisfactory results were obtained: descriptive variables (*k* = .98), interpretive variables (*k* = .84). S2 Appendix contains a complete list of the academic articles included in this study, while S3 Appendix lists the relevant policy documents by region.

### 3.3. Diffusion patterns of central concepts–overview of the academic debate

Fig 1 shows the distinct occurrences of the terms ‘public engagement’ and ‘science’ together in either titles or abstracts (not necessarily both) of publications in the WoS for the period 1995–2019 (N = 517). As the graph demonstrates, the co-occurrence of the two terms first became common in 2003, with subsequent significant increases in 2009, 2011, 2014 and 2017.

Fig 2 shows the top ten journals ranked according to the number of publications with ’public engagement’ and ’science’ in titles or abstracts. Overall, 273 journals from the WoS were found containing the combination of these terms in titles or abstracts. The numbers indicate, at least broadly, that journals specializing in science communication (*Public Understanding of Science* and *Science Communication*), as well as science policy and STS (*Science and Public Policy*, *Science*, *Technology & Human Values* and *Science as Culture*) frequently publish on this topic.

**Fig 3:**
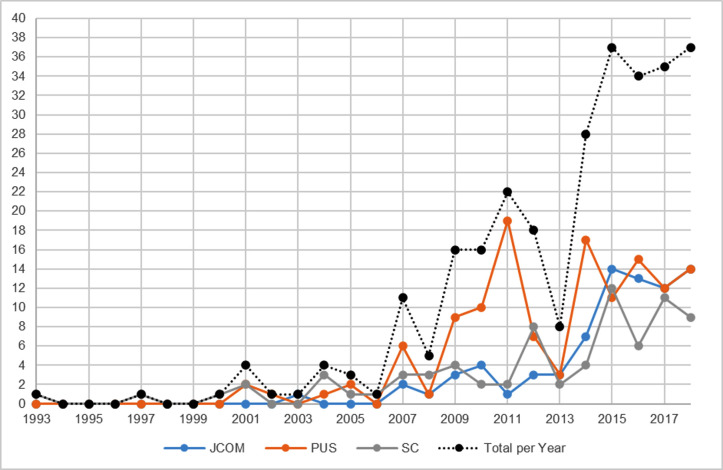
Occurrences of the term ‘engagement’ in published items in *JCOM*, *PUS* and *SC* in titles and/or abstracts (1993–2018; N = 284).

When considering the three journals under investigation in our study, namely, *JCOM*, *PUS* and *SC*, a similar increase in the various forms of the term ‘engagement’ can be noted in 2009 and 2011, with a further spike in 2015. Fig 3 demonstrates the occurrences of ‘engagement’ in the titles and/or abstracts of articles in these three journals for the period 1993–2018 (N = 284).

**Fig 4:**
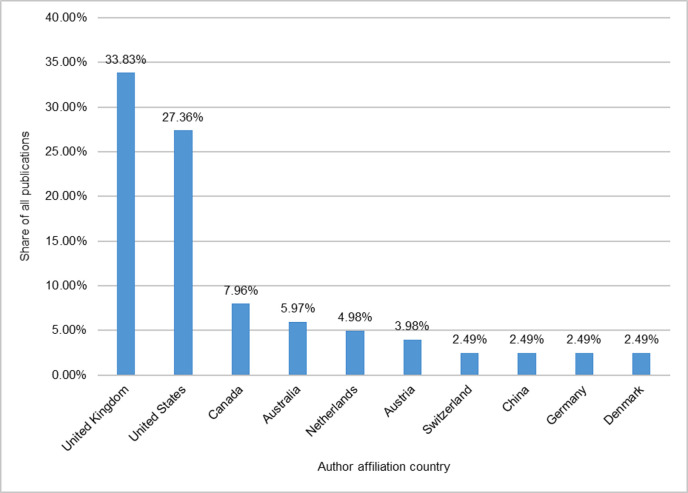
Country affiliations of authors in the project sample who published in *PUS* and *SC*, as captured by the Web of Science (N = 206).

The country affiliations of authors are another indication of the origin and location of the discourse on engagement. It is particularly the UK and the USA that show a high presence in the case of the set of publications identified for this study (Fig 4). Patterns of country affiliations are similar for articles published in *PUS* and *SC* in our sample (N = 206) and published items captured in the WoS (N = 517).

## 4. The academic discourse on engagement

In the following section, examples from journal articles in *JCOM*, *PUS* and *SC* are provided for our first three RQs: definitions, motivations, and criticisms of ‘public engagement with science’. In the case of motivations and criticisms, codes are illustrated with examples.

### 4.1. How is public engagement with science defined in academic literature?

Our first RQ focused on explicit or implicit definitions of engagement. Within the sample of academic articles analyzed (N = 86), only six provided an explicit definition for engagement, while a further 35 contained a phrase providing some indication of what the author(s) meant by engagement. This means that in more than half of the sample (n = 45) no explicit or implicit definition was provided.

The concept of public engagement with (or in) science has multiple origins which result in a wide range of conceptualizations of the term. There are also multiple models for its implementation, encompassing a considerable variety of activities that are aimed at many different audiences. Public engagement is described as “an umbrella term” [51, p. 557], a “catch-all” term and a “buzzword” [1, p. 242–244]. According to Rowe et al. [52, pp. 420–421], “engagement is not a simple concept”, because of the multiple reasons for conducting engagement and various methods for achieving this. Moreover, scientists have multiple definitions of public engagement in mind when they participate in science engagement activities and the “definition of the term engagement remains an area of both academic and practical debate” [53, p. 392].

#### 4.1.1. Defining engagement in terms of its objectives

Definitions can also be *implied* in the objectives of public engagement with science, e.g., in terms of achieving “greater visibility and transparency of academic work and increased academic accountability” [54, p. 754] or “bringing inclusiveness, transparency, diversity and creativity into the research and innovation process” [55, p. 738]. This is differentiated from the objectives of more traditional science communication formats that were practiced for “mere entertainment or cultural purposes” [56, p. 2].

Seeking “lay people’s opinions on new techno-scientific possibilities” [57, p. 153] is another objective, although this objective may be driven by a desire to increase public acceptance of new technologies. Rowe et al. describe public engagement of science as “something of an antidote to difficulties associated with the traditional manner of setting policy” [58, p. 353]. Nava and Hofman refer to the objectives to “reduce conflict” and “build trust”, leading to publics who are then “more likely to support project goals and implement decisions in the long term” [59, p. 4].

#### 4.1.2. Defining engagement in terms of intended audiences

Science engagement is also defined implicitly in terms of the audiences addressed. They are meant to be “non-scientific” [60, p. 3] or “individuals who are not associated with any scientific discipline or area of inquiry as part of their professional activities” [61, p. 2], “hard to reach voices that are commonly excluded from public debates" [62, p. 208], and children [63].

#### 4.1.3. Defining engagement in terms of a new role for the public

From the early 2000s onward, an increased emphasis on the fact that citizens should have a say in decisions about science, can be noted. This is attributed not only to their democratic rights, but also to the public’s potential expertise and experiences that could enrich and inform science [64–66]. The role of citizens should enable “non-experts to become the actual protagonists in the scientific decisions producing social effects” [67, p. 1]. The involvement of citizens should allow them to play a role in both planning of new policies and decision-making about policies [52, 68]. Indeed, ideas concerning the importance of citizens’ input has become so commonplace that seeking the input of lay people on emerging technologies is described as “standard procedure” [57, p. 153].

#### 4.1.4. Defining engagement in terms of the nature of the relationship between science and the public

Since the early 2000s, descriptions of the nature of science engagement have an explicit focus on its dialogic (or interactive) character that involves multiple stakeholders [69] with an information current that flows both ways between scientists and the public [70]. Riesch, Potter and Davies note that “conceptions of public engagement have shifted over the years as research on public understanding of science has moved through its various paradigm shifts from deficit to dialogue to upstream engagement and ‘third mode’ engagement and finally attempts to reconcile them” [71, p. 2]. The relationship is described as a “meaningful conversation and dialog about scientific issues” [61, p. 2] that should involve a genuine interactive engagement between scientists, stakeholders and the public [72, p. 513]. Furthermore, “symmetry in learning” is highlighted as an indicator of true public engagement [73, p. 809].

In an effort to make it clear that engagement is something different from public understanding of science, Perié et al. explicitly state that engagement does not imply simplification of scientific knowledge for mere entertainment or cultural purposes. Rather, it entails sharing the essence of the production of this knowledge that is meaningful and relevant, especially when engaging “people living in deprived areas” [56, p. 2]. Jia et al. add that effective public engagement “means figuring out ways to structure and promote conversations with the public that recognize, respect, and incorporate differences in knowledge, values, perspectives, and goals” [74, p. 5]. Moreover, Davies describes public engagement as a relational activity that is about “building bridges, crossing gaps, creating connections, partnering, enabling mutual benefit, facilitating relationships, or breaking down barriers” [75, p. 694].

#### 4.1.5. Defining engagement in terms of type of activities

Engagement is also defined in terms of a wide range of activities designed to achieve it, as can be seen in the following examples:

“[…] appearing on radio, giving a public lecture, designing activities for children, and so on” [76, p. 244].

“[…] public or school conferences, appearances on radio, interviews in newspapers, written work for non-specialist audiences, participation in open days or science festivals, collaboration with associations, design of activities for children, and so on” [77, p. 54].

“[…] lecturing in public or in schools, giving interviews to journalists for newspapers, radio or television, writing popular science books, writing the odd article for newspapers or magazines oneself, taking part in public debates, volunteering as an expert for a consensus conference or a ‘café scientifique,’ collaborating with non-governmental organizations (NGOs) and associations as advisors or activists, and more” [60, p. 3].

“[…] it can take the form of one-way attempts to provide content through the news media, advertising, Internet sites, or presentations, to more interactive activities where participants are invited to participate in two-way dialogue” [78, p. 653].

#### 4.1.6. Engagement as ‘upstream technology assessment’

One source of the discourse on ‘engagement’ is ‘upstream technology assessment’, the idea that the public should be involved at an early stage in the development of new technologies. Rogers-Hayden and Pidgeon define ‘upstream engagement’ as:

“Dialogue and deliberation amongst affected parties about a potentially controversial technological issue at an early stage of the research and development process and in advance of significant applications or social controversy” [79, p. 346].

Similarly, Krabbenborg and Mulder describe upstream public engagement as follows:

“[…] a new governance vision in which citizens and civil society organizations right from the early stages of research and development trajectories, engage in dialogue with technology developers, such as scientists and industrialists, about the (tacit) assumptions, meanings, values, and consequences of new science and technology for society” [80, p. 453].

Notwithstanding its noble intentions, Rogers-Hayden and Pidgeon point out that “the notion of upstream engagement is a contested concept with a range of associated dilemmas and tensions” [79, p. 345].

### 4.2. Key motivations attributed to public engagement with science in academic literature

Answering RQ2, public engagement with science is motivated in various ways. Within the sample of academic articles analyzed for this study (N = 86), the majority (n = 72) mention a motivation or motivations for public engagement with science. The most pertinent motivations are democratization, education, legitimation, innovation and inspiration. Table 2 provides our definition for each of these motivations alongside the number of occurrences, and percentage of the total, for each motivation during two time-periods, 2000–2009 and 2010–2018. We chose to divide the time-periods in this way in order to gain a clearer picture of the growth of motivations over time. As explained above, (see section 3.3 and Fig 1) the first significant increase in the co-occurrence of the terms ‘public engagement’ and ‘science’ occurred in 2009. Therefore, we chose to end the earlier period in that year. Since many articles had more than one motivation, the total below exceeds 86. When considering these numbers, an increase in motivations during the later time-period is evident.

**Table 2:** Primary motivations for engagement in academic literature.

Motivation	2000–2009	2010–2018
**Democratization**: engaging to empower citizens to participate competently in society (democratization of society) and/or to participate in science (democratization of science)	5	28
	5.8%	32.6%
**Education**: engaging to inform and educate the public about science, improving (general or specific) public access to scientific knowledge	8	24
	9.3%	27.9%
**Legitimation**: engaging to promote public trust in and acceptance of science, as well as policies supporting science	10	18
	11.6%	20.9%
**Innovation**: engaging to promote innovation, the public or citizens are considered to be a valuable source of knowledge (e.g. local expertise) and are called upon to contribute to knowledge production, bridge building and including knowledge outside ‘formal’ science	3	19
	3.5%	22.1%
**Inspiration**: engaging to inspire and raise interest in science, to secure a STEM-educated labor force	2	14
	2.3%	16.3%

#### 4.2.1. Democratization

Engaging to empower citizens.

In coding, we did not differentiate between the two possible interpretations of democratization, i.e. to participate competently in society (democratization of society) and/or to participate in science (democratization of science). They occurred in different combinations. These are pertinent examples:

“There is a strong democratic principle that citizens will be better able to contribute to decisions that are likely to have an impact on their lives and become engaged with the decision-making process” [53, p. 368].

“A shift from public understanding to public engagement with science has been characterized as citizen-oriented science or a more open, egalitarian, and participatory science. These descriptions aim to acknowledge the importance of citizens’ concerns and perspectives and consider the dialog between scientific and citizen groups to be a crucial element of the modern ways of knowledge production and governance” [61, p. 1].

“Diversity is an important principle of deliberative democracy on which public engagement activities should be based […] The scholarship on public engagement in the context of Western liberal democracies in recent times has acknowledged the need to ‘hear all voices’/include multiple perspectives. Yet, in the realm of science and technology, this goal still falls short of what is in fact needed–rethinking the very architecture of decision-making processes on science policy so that it truly embodies cultural diversity” [81, p. 288].

#### 4.2.2. Education

Engaging to inform and educate the public about science, improving (general or specific) public access to scientific knowledge.

Assessments as to how much prior knowledge is needed for engagement, and if providing such knowledge should be an objective, vary.

“Our findings suggest that little prior formal science knowledge is necessary in a debate of this kind […] Particularly for the active/generative participants, what was needed was a critical understanding of the nature of scientific evidence and a grasp of the way that wider issues influence debates about science and the value that is placed on the formal scientific evidence. In terms of the science curriculum, it follows that there is a need to teach for an understanding of scientific evidence and for pupils to actively engage with topical science-based issues” [82, p. 362].

“The general public should be enabled to understand complex systems, uncertainty, statistics, and the difference between science and pseudoscience” [83, p. 290].

“Once engagement was initiated, our work helped build citizens’ knowledge about nanotechnology, as well as their confidence and efficacy levels to understand and engage with it—which in turn built their capacities to engage further with other citizens, scientists, and policymakers” [84, pp. 131–132].

#### 4.2.3. Legitimation

Engaging to promote public trust in and acceptance of science, as well as policies supporting science.

Legitimation as a motive to promote engagement aims at the acceptance of science (and technology) in general.

“The shift from merely promoting the understanding of science–as indicated by PUS–to emphasizing the need for public engagement is seen as necessary to obtain public confidence in science” [67, p. 1].

“Engagement provides an ‘antidote’ to pathologies associated with a ‘deficit-model’ approach to decision-making; […] making decisions without public support is liable to lead to a number of practical difficulties, such as confrontation, disruption, boycott, and public distrust. Indeed, a decline in trust in policymakers has been widely noted and is regarded as having compromised the perceived legitimacy of governance in some areas of policy development. A transition seems to have occurred from a position where information was seen as the key to resolving a knowledge deficit, and so resolving lay opposition, to one in which regaining trust in governments and regulators is seen as vital to solving a perceived legitimation (or trust) deficit” [85, p. 332].

#### 4.2.4. Innovation

Engaging to promote innovation.

One motivation for engagement is that it will increase innovation. We included in this the argument that the public, i.e. citizens, are a valuable source of knowledge (e.g., local expertise) and should be called upon to contribute to knowledge production. Here the emphasis is often on bridge building and including knowledge outside ‘formal’ science.

“Local community engagement and support is seen as key to the continued development and the increasing deployment [of renewable energy technologies]” [86, p. 37].

“Lay citizens have valuable knowledge and perspectives to bring to discussions about technological developments, and their input can contribute to more comprehensive and robust societal decisions about these developments” [84, pp. 131–132].

#### 4.2.5. Inspiration

Engaging to inspire and raise interest in science, to secure a science, technology, engineering, and mathematics (STEM) educated labor force.

“The program aimed to inspire a new sense of excitement amongst young people around the physical sciences. […] The data show that children as young as 10 are thoughtful about their futures, and may already be forming strong opinions. Indeed one might say that science engagement initiatives that support and target primary age students would not be wasting their time” [87, pp. 1, 3, 13].

“Another purpose of public engagement initiatives is to reverse the decline of science as both an academic subject and a profession […] This leads many public engagement initiatives to focus on directly improving the attitudes of young people toward science as an academic subject rather than improving attitudes toward science in general” [65, pp. 178–179].

### 4.3. Key criticisms or concerns about public engagement with science in academic literature

Within the sample of academic articles analyzed for the current study (N = 86), the majority (n = 68) mention some form of criticism or concern about public engagement with science (RQ3). Table 3 provides a description of some of the major criticisms related to engagement or its implementation alongside their number of occurrences, and percentages of the total. As before, the results are split between 2000–2009 and 2010–2018 to highlight change over time. Since many articles had more than one criticism, the total below exceeds 86. Again, an increase in the various criticisms during the later time-period is apparent.

**Table 3:** Prominent criticisms of engagement in academic literature.

Criticism	2000–2009	2010–2018
**Practical limitations** of engagement: e.g. lack of time and resources, constraints of group size	7	14
	8.1%	16.3%
**Evaluation:** shortage of evaluation conducted and or difficulties in operationalizing evaluation	8	9
	9.3%	10.5%
**Scientists are not prepared/trained** for engagement: being in the public eye or addressing the general public runs counter to the established norms of science	7	9
	8.1%	10.5%
**Deficit model** persists beneath engagement rhetoric	5	8
	5.8%	9.3%
**The true intentions of engagement**: public views should have impact, effect decision-making and be more than a mere tokenistic gesture	3	9
	3.5%	10.5%
**Diversity of audiences and their needs:** a ‘single public’ for engagement does not exist	0	7
	0%	8.1%
**Factors that deter scientists from getting involved in engagement:** lack of incentives for scientists to participate; lack of recognition and professional reward; engagement considered at odds with an academic career and not well-regarded in the scientific community; lack of institutional support	3	7
	3.5%	8.1%

#### 4.3.1. Practical limitations of engagement

The most frequently identified concern relates to the practical limitations of engagement. This includes a lack of time and resources, as well as constrains related to group size. Time constraints, in particular, are noted as a concern regarding engagement for both individuals participating in engagement activities and organizations or institutions involved in facilitating engagement, as the quotes below illustrate:

“It is worth asking how average citizens will find the time to be civically engaged, and under what conditions they will be inclined to do so” [88, p. 223].

“While some civic society organizations had initiated and designed public dialogue processes, many lacked the resources (time and money) to do this” [89, p. 291].

#### 4.3.2. Concerns related to the evaluation of engagement

The importance of evaluation in making any statements about the efficacy or impact of engagement is repeatedly highlighted. Rowe, Poortinga and Pidgeon emphasize that systematic evaluations are rare but essential if “unsupported contentions as to when any particular approach might or might not be useful” [58, p. 354] are to be avoided. Longstaff and Secko point out that, despite broad recognition of the importance of evaluation as a vital step in improving practice, evaluations are rarely conducted [90, p. 252]. Without effective evaluation of real-world examples of public engagement activities, any benefit or impact remains difficult to determine [85, p. 332]. Moreover, even in cases where evaluation is done, the results often remain unpublished for a variety of reasons [91].

A number of practical challenges in evaluating engagement are acknowledged, such as ambiguity over how to operationalize evaluations [90], the need for a more scientific approach [92], as well as a larger evidence base. However, the general sentiment remains that more frequent and effective evaluation is essential and engagement without evaluation is of limited value [54, p. 755]. There appears to be general agreement that, despite its challenges, evaluation should play a central role in engagement and not “be placed on the shelf as an ivory tower ideal, only to be dusted off on rare occasions when an academic comes around with mounds of spare time and resources” [92, p. 4].

#### 4.3.3. Scientists are not prepared or trained for engagement

Training is seen as an essential pre-condition for mobilizing (at least some) scientists to take part in effective public engagement [93]. Communication skills generally do not feature in the training of scientists [65, 78, 83]. Some authors recognize that engagement training for scientists is becoming more commonplace, but there are still many reasons why scientists do not pursue this kind of training:

“While numerous organizations now offer training for ‘engagers’ seeking to develop their skills, pressures of time and organizational and peer support can result in many developing such attributes via experience alone” [53, p. 392].

#### 4.3.4. The persistence of the deficit model

A repeated concern relates to the persistence of the deficit model approach to science communication underneath ‘engagement’ rhetoric. This is explained by the fact that scientists mostly continue “seeing the citizen as an empty vessel to be filled with scientific knowledge” [83, p. 289]. Wilkinson, Bultitude and Dawson point out how culturally entrenched deficit-style thinking has become:

“Despite the ‘grand narrative’ of public engagement among a variety of organizations, much talk around more participatory engagement activities is entrenched by cultural habit around notions of public understanding which will not evaporate rapidly” [53, p. 391].

Rogers-Hayden and Pidgeon cite one respondent’s apt summary of this situation:

“[…] it’s a kind of deficit model again, a deficit model of public engagement—we’ll dialogue in order so that they understand us better and will agree with us, instead of we’ll sit them down and educate them” [79, p. 355].

Ellis, Waterton and Wynne explain how the danger in this lies in the possibility of silencing the voices of the very publics with whom engagement is sought:

“If public engagement exercises remain stuck in a deficit style of science communication, this will ‘ironically’ help to ‘reinstate the authority of science by subtle means involving erasure of the very publics being invited to participate’” [94, p. 504].

#### 4.3.5. The true intentions of engagement

“Are engagement efforts really intended to involve citizens in ways that could give them a meaningful voice in science and technology decision-making? Or is the goal to increase citizens’ trust in scientists and policymakers or to encourage them to accept nanotechnology products? Relatively few engagement projects, for example, include processes intended to link citizens’ recommendations, concerns, and questions to actual policy processes or decision-makers” [84, pp. 128–129].

The question of whether public input is taken seriously and how it feeds into science policy is mentioned several times. A similar criticism relates to the ‘true’ motives behind ‘public engagement’. These authors question whether engagement is just “a form of risk prevention, meant to secure public acceptance” [1, p. 245], or whether a “compliant” public is sought despite “proclamations of public deliberation and publics’ scientific citizenry” [54, p. 770]. Stilgoe, Lock and Wilsdon even question whether public engagement practices are “used to close down vital debates in contentious areas” [95, p. 11].

#### 4.3.6. Diversity of audiences and their needs

Several authors emphasize the importance of taking audience diversity (and consequently diverse needs) seriously and avoiding the notion of a single public [55, 73, 86, 94, 96]. Munshi et al. for example, highlight the need “to go beyond a generic notion of public engagement that assumes the public to be one homogeneous category” and warn of the dangers of tokenistic engagement with minority groups [81, p. 288].

#### 4.3.7. Factors that deter scientists from getting involved in engagement

Lack of institutional support and recognition are highlighted as key factors that deter scientists from getting involved in public engagement:

“[…] it is a culture of public engagement that still seems to be lacking among most research institutions in Europe–meaning an organizational culture in which PE is appropriately recognized, evaluated and rewarded as part and parcel of the organization life, routine activities, and identity as well as a relevant element of the broader institutional landscape in which the organization operates” [93, p. 77].

Other deterring factors include the fact that public engagement activities are viewed as being “at odds with traditional academic career paths and reward systems” [65, p. 178], lack of incentives [97] and the notion that engagement about one’s own research is self-promotion [74].

## 5. The science policy programs on public engagement with science

Our sample of policy documents is too small to allow the construction of a timeline of the development of the ‘engagement’ rhetoric in the policy arena. It is indicative, however, that neither the Bodmer Report of 1985, nor the Wolfendale Report of 1995 mention the term ‘engagement’ at all. An indirectly related analysis by Conceição et al. for the occurrence of the term ‘public’ in EU framework programs shows a cycle beginning in FP 5 (1998–2002), morphing to ‘society at large’ and being replaced by ‘all stakeholders’ in H2020 (2014–2020) [15, p. 17].

### 5.1. How is public engagement with science defined in policy documents?

Some policymakers attempt to provide a catch-all definition by describing engagement as “an overarching term” that includes science, science communication, science literacy and science outreach and awareness [98, p. 13]. Similarly, a wide range of diverse activities is seen as forming part of public engagement with science, including “science festivals, centers, museums, cafes, media, consultations, feedback techniques, and public dialogue” [26, p. 19].

Several definitions emphasize “mutual learning” and “dialogue” between experts and public audiences [98, 99]. Similarly, engagement is defined as “seeking and facilitating the sharing and exchange of knowledge, perspectives, and preferences between or among groups who often have differences in expertise, power, and values” [100, p. 35].

The objective of ensuring public acceptance of new technologies, although expected to be very important to policymakers, is rarely explicitly mentioned in policy documents as a key objective for public engagement. EU policymakers suggest a tentative link between public engagement and public support by saying that it “offers the possibility of fostering new forms of public appraisal and maybe appreciation of research and technology development” [101, p. 4].

It is specifically stated that the aim is “not about promoting blind faith in science or an unquestioning acceptance of its authority, but rather about providing everyone with the understanding and the opportunity to contribute to debates when science is discussed” [26, p. 28]. There are, however, some indirect references to the need to nurture public support, for example, the strengthening of communications regarding broader impacts success was seen as “critical to public and political perception of and support for science” [102, p. 7]. In general, policymakers tend to emphasize that the objectives of public science engagement are for the public good. For example, this UK definition states the following four key goals: (1) engaging under-represented communities; (2) actively involving a wide range of people; (3) nurturing a future generation passionate about research and innovation, and (4) listening to public concerns and aspirations [20]. The South African Department of Science and Technology (DST) (renamed the Department of Science and Innovation (DSI) in 2019) combines the promotional goal of “popularizing” and “promoting” science [98, p. 3] with the democratic goal of developing a “critical public that actively engages and participates” in science [98, p. 3].

Policy documents are mostly vague about the public audiences that should be targeted for engagement, and what is expected of them. Reference is made to “a distinct role for citizens or stakeholder groups in research and innovation processes” [103, p. 1] or “creating opportunities for people to discuss, create and participate in research and innovation” [20, p. 2]. Public audiences are involved to “varying degrees” at contributory, collaborative and co-creative levels [104, p. 7]. In general, researchers are reminded to think of public engagement as “communicating *with* the public rather than *at* the public” [105, p. 4], and to move away from “communication of research findings” to “active participation” with the public [20, p. 4].

### 5.2. Key motivations attributed to public engagement with science in policy documents

The central motivations for public engagement in policy documents (RQ2) are democratization, education, legitimation, innovation and inspiration (the same as in the academic discussion above). Some policy documents emphasize the value of public engagement for its potential to include societal knowledge into deliberations, thereby broadening the knowledge base for policymaking and nurturing public trust in science [106].

In addition to the functional and political motives of public engagement, policymakers also highlight its cultural motives, including that lay people move from “outsiders” to “co-creators” where they have a say and can participate in science, resulting in a more equitable relationship between science and society [101]. The need to value and respect the views and specific knowledge that public groups can bring to every step of the scientific process is also emphasized. Examples for each of these motivations, for each of the four regions under discussion, are provided in Tables 4–8.

**Table 4:** Democratization as motivation for engagement in policy documents.

Democratization: engaging to empower citizens to participate competently in society (democratization of society) and/or to participate in science (democratization of science)			
UK	USA	EU	SA
“It is vital that the public have both access to the knowledge research generates and the opportunity to influence the questions that research is seeking to address. In enriching citizenship and providing wider perspectives on research, public engagement improves the quality of research” [107, p. 1].	“In a democracy, managing public goods requires active civic engagement to sustain these resources and ensure their equitable distribution and public access. By engaging in such acts as deliberation, persuasion, and the donation of time and money, members of the public participate both in decisions about the use of scientific knowledge (e.g., ways of minimizing air pollution) and decisions about the allocation of resources to the production of scientific knowledge (e.g., supporting funding of stem cell research)” [108, p. 22].	“A boost in democratic legitimacy, accountability and transparent governance can be one of the main positive outcomes, especially for an institution such as the European Commission often seen as not being close to citizens” [104, p. 3].	“It is important to ensure that science and technology serve society by enabling citizens to engage in debate around matters of public interest that are scientifically or technically complex” [98, p. 6].

**Table 5:** Education as motivation for engagement in policy documents.

Education: engaging to inform and educate the public about science, improving (general or specific) public access to scientific knowledge			
UK	USA	EU	SA
“[…] all cultural organizations have the power, the potential and the responsibility to help engage, inspire and educate a generation of young people and an occasionally skeptical wider public in science” [26, p. 18].	“[Some] goals are best achieved by the dialogue that occurs through formal public engagement. Such goals as generating excitement, sharing information needed for a decision, and finding common ground among diverse stakeholders all lend themselves to public engagement as a communication strategy” [100, p. 35].	“Public engagement initiatives are faced with barriers […]. The PE2020 project identified a plethora of these barriers […] [including] scarce education before consultation on PE […]” [103, p. 3].	“In the current global ‘post-factual’ society, raising science awareness is of increasing importance in efforts to provide credible alternatives to dubious sources of information” [109, p. 72].

**Table 6:** Legitimation as motivation for engagement in policy documents.

Legitimation: engaging to promote public trust in science and science policy processes			
UK	USA	EU	SA
“[…] the crisis of trust has produced a new mood for dialogue. In addition to seeking to improve public understanding of their work, scientists are beginning to understand its impact on society and on public opinion” [18, p.2].	“The process of public engagement can help build and sustain trust among stakeholders and aid in finding common ground […]. What is known now […] is that public engagement often is essential for acceptable decisions about science-related controversies” [100, p. 30].	“The quality, capacity and legitimacy of European science, technology and innovation need to be boosted. In order to do this, new governance tools and working methods are needed for public and societal engagement of science” [110, p. 1].	“Profiling South African science, technology and innovation […] in order to enhance South Africans’ appreciation of the value of science, to lobby for public support for government’s continuous investment in science and technology, and to allow the public to hold government accountable for using science for the public good” [99, p. 30].

**Table 7:** Innovation as motivation for engagement in policy documents.

Innovation: engaging to promote innovation, the public or citizens are considered to be a valuable source of knowledge (e.g. local expertise) and are called upon to contribute to knowledge production, bridge building and including knowledge outside ‘formal’ science			
UK	USA	EU	SA
“As the pace of innovation continues to increase, it is more important than ever that policymakers, funders, researchers and innovators are able to engage society in the development of plans and priorities” [20, p. 6].	“[…] broader impacts are an essential component of the work of NSF as an innovation agency at the frontiers of knowledge” [105, p. 5].	“Public engagement produces new resources, enhances capacities and […] provides actors outside academia access to the production process of scientific knowledge and hence the possibility to utilize the knowledge already during the process itself” [110, p. 2].	“[…] we must ensure that our citizens are aware of the importance of science for the growth of the economy and the well-being of ordinary people, and are sufficiently informed about science to engage critically with policymakers [98, p. 2].

**Table 8:** Inspiration as motivation for engagement in policy documents.

Inspiration: engaging to inspire and raise interest in science, to secure a STEM educated labor force			
UK	USA	EU	SA
“There is beginning to be a tangible impact in terms of interest among potential students about attending the university to take degree courses” [111, p. 8].	“Some goals of science communication […] are best achieved by the dialogue that occurs through formal public engagement. Such goals as generating excitement, sharing information needed for a decision, and finding common ground among diverse stakeholders” [100, p. 35].	“[…] at least part of the aim here is to encourage the students to think about science as a potential career” [112, p. 103].	“Scientific literacy is important for the maintenance and expansion of national systems, because it can enhance the appeal of science as a career and as a social knowledge system” [98, p. 6].

### 5.3. Key criticisms or concerns about public engagement with science in policy documents

Parallel to the coding of the academic articles, we also coded for critical appraisals of public engagement with science in policy documents. We expected that, by virtue of their nature as policy directives in favor of science engagement, these types of documents would be less critical of public engagement with science. Nevertheless, the majority of policy documents (13 of the 19) mention some form of criticism or concern about public engagement with science. Examples from the different regions are discussed below.

Concerns regarding public engagement with science mentioned in policy documents are surprisingly similar to those pointed out in academic articles. They frequently relate to the lack of time, resources, infrastructure and skills to enable effective engagement practice, as well as the need for a new pro-engagement culture within research organizations [106], so that engagement can become “mainstreamed” as an integrated part of research [113].

#### 5.3.1. Need for support, recognition and reward

Other common concerns relate to the necessity of greater recognition of the value of public engagement, as well as greater support and reward for public engagement work in scientists’ career context; see Table 9 for pertinent examples.

**Table 9:** Concerns mentioned in policy documents: Need for support, recognition and reward.

Need for support, recognition and reward			
UK	USA	EU	SA
“There is scope for making public engagement […] perceived as more professional in its approach and a valued part of the work of scientists. There is more need for […] the best in public engagement to be incentivized and rewarded” [26, p. 20].	“Universities need to demonstrate their commitment to broader impacts and provide greater incentives for people to promote broader impacts” [105, p. 5].	“The need for engagement should be made explicit in calls for proposals.” Furthermore, “scientists need to be rewarded for engaging actors affected by their research and innovation activities” [113, pp. 3–4].	“Scientists who participate in science engagement activities will be awarded continuing professional development points […]” [109, p. 56].

#### 5.3.2. Scientists are not prepared or trained for engagement

Scientists’ general lack of preparedness to engage with public audiences is a further prominent criticism, raised in six of the 19 policy documents; see Table 10 for pertinent examples.

**Table 10:** Criticisms in policy documents: Scientists’ lack of preparedness.

Scientists are not prepared or trained for engagement			
UK	USA	EU	SA
“Research Councils and universities should strongly encourage communication training for scientists […]. The communication training offered to research students should be broadened to include an awareness of the social context of their research and its applications […]” [18, p. 3].	Public engagement does not come easily to most scientists; i.e. “it is an acquired skill” [100, p. 24].	“The skills needed to run meaningful and successful citizen engagement processes are to be addressed by building in-house capacity, concerning for example tools, guidance and complementary training” [104, p. 4].	The South African DST is explicit about the need to train scientists and researchers in science communication and science engagement skills, adding that: “[…] these trained researchers and scientists would then help to introduce developmentally appropriate engagement activities and projects for both adults and school learners” [109, p. 56].

#### 5.3.3. Lack of credible evaluations

Policymakers recognize that evaluation is critical to improve engagement approaches, to avoid pitfalls and to provide evidence of its value and impact over time [113]. The lack of publicly available data on credible and robust evaluations of public engagement activities surfaces in several policy documents, and policymakers call for sound evidence of good practice in the field [106].

#### 5.3.4. Practical limitations of engagement

A final criticism worth noting in policy documents is the practical limitations of engagement, including lack of time and resources as well as constraints of group size. These are mentioned in six of the 19 policy documents. Coincidently, all of these are EU policy documents. These policymakers acknowledge the complex challenges of effective public engagement with science by stating, for example: “The question now seems not to be whether public involvement should occur, there is a great deal of normative argumentation for doing it; the questions seem to be now about how it should occur, at which point of the process, its impacts and in what fields is it legitimate and relevant. Notwithstanding the more accommodating tendency, effective public engagement exercises are full of difficulties of theoretical (what is effectiveness in this context?), practical (how do we assess that?) and of political (how can this be done in often contested terrains?) natures” [112, p. 15]. It is also recognized that “engagement processes often fail for a number of reasons, including insufficient preparation of participants and decision-makers or insufficient consideration of how the outputs of the process would be taken forward” [114, p. 2].

## 6. Discussion

Our initial assumption about the nature of the engagement rhetoric is corroborated by the detailed content analysis: the vagueness amongst science communication scholars and science policymakers regarding the most appropriate formats, features and objectives of public engagement with science is striking. It is apparent in the virtual absence of any clear definition of what ‘engagement’ is supposed to mean. The characterizations as “an umbrella term” [51, p. 557] and “an overarching term” [98, p. 14] in both the academic and political rhetoric, amount to an effective surrender to the plethora of meanings, interpretations and activities that are all seeking inclusion in the popular appeal of the buzz. ‘Engagement’ is sometimes used interchangeably with ‘participation’, but we have not captured such documents on the same scale. In fact, the literature on forms of ‘participation’ is more specific, typically dealing with case studies, but the term ‘engagement’ is more ambitious while inevitably also becoming more general [115].

The vagueness of definitions of ‘engagement’ is further reflected in the virtual non-existence of specific concepts of the ‘public(s)’ to engage with science, other than alluding to “citizens or stakeholder groups” [103, p. 1], or “non-scientists” [61, p. 2]. This is likely an unavoidable consequence of the paradoxical construction of the ‘engagement’ project: inviting participation–short of conceding the ‘real’ power of decision-making–must remain indeterminate.

The lack of clear definitions is also mirrored in the wide-ranging and varied objectives of public engagement. They range from “mere entertainment” [56, p. 2] to building trust [59, p. 4] and “bringing […] creativity into the research and innovation process” [55, p. 738]. In other words, the abstract public is seen as either a passive audience, a source of support and legitimacy, or a source of ideas. ‘Legitimation’ as an objective is prevalent in policy documents, and prominent even in academic articles. In connection to this, it is expedient to point to the origins of the engagement rhetoric in the context of dealing with the BSE crisis in the UK as evidenced by the Bodmer [116] and House of Lords [18] reports, and the overwhelming dominance of authors from that country in the pertinent academic journals (see Fig 4). Evidently, public engagement with science was first and foremost a British issue before it began to spread.

The functions of legitimation and democratization are interconnected in the sense that in order to obtain a legitimating effect some democratizing measures have to be implemented. However, they remain specious if they are ‘just’ supposed to have a legitimating function. While the South African White Papers of 1996 [117] and 2019 [109] postulate an informed public as an asset to the National System of Innovation, the EU expects from engagement in the abstract “a boost in democratic legitimacy, accountability and transparent governance” for the European Commission [104, p. 3].

Our data do not allow us to reliably reconstruct a shift over time regarding the relative weight assigned to ‘education’, ‘democratization’, ‘legitimation’, ‘innovation’ and ‘inspiration’ as motivations. Nonetheless, the comparison between academic articles and policy documents lends support to the conjecture that the dynamic of the rhetoric reveals a pattern: ‘education’ as a motive was denounced in the academic discourse as a ‘deficit-model’ to be overcome by a ‘democratization’ of science, the true dialogue between science and the public. This argument appealed to policymakers for its legitimating value, but with a significant shift in emphasis on the potential value of the public’s input for the promotion of innovation and creating a workforce in STEM fields. This is in line with findings for the policy discourse in the EU by Macq, Tancoigne and Strasser [8]. The perfunctory nature of the ‘engagement’ project is also underscored by the observation that the objective of getting public input, i.e. ‘engaging’ with the public, is hardly ever operationalized. There is a lack of descriptions of meaningful and sustainable mechanisms to achieve this objective. Nor is there a clear definition of particular groups or stakeholders whose needs could be met, as is, for example, the case in health communication. Instead, most case studies refer to ad hoc groups such as visitors at public science events or places such as science centers [82, 118, 119]. This has its equivalent in the effective impossibility of evaluation. Programs whose essence remain undefined, whose objectives are varied and equivocal and whose practicalities are absent cannot be evaluated to assess their effectiveness.

The lack (or impossibility) of evaluation is a prominent criticism among those raised by academic authors, but not the only one. They also complain about the unpreparedness of scientists, various practical obstacles and–more unmasking–the persistence of the deficit model. The policy documents, although they are mostly programmatic, sound the same criticisms. With rare realism, an EU policy states that “[the] model of knowledge co-production where citizens and concerned groups get actively involved in the process of knowledge production is still far off” [112, p. 10], and a 2016 USA policy diagnoses “the work of science is complex: it is a process, a product, and an institution. As a result, engaging in science—whether using knowledge or creating it—necessitates some level of familiarity with the enterprise and practice of science; we refer to this as science literacy” [108, p. 22].

The fourth research question concerned the connection between the academic and the political discourses. Irwin commented on this relationship as early as 2006: “In remarkably few years, an (admittedly attenuated) form of the language of STS has been reconstructed as the language of policy. Without wishing to attribute uni-linear causality […] it is certainly possible to identify a resonance–and at times an explicitly-drawn connection–between previous STS research and the current policy emphasis on trust, transparency, uncertainty and dialogue” [41, p. 300]. Unfortunately, the level of our analysis and the resolution of our instrument, i.e. the codes, do not allow for making inferences about causal relationships. The evidence of such a relationship lies in the fact that the terminology employed is virtual identical and the reasoning this communicates.

However, other sources prove–at least for the EU–that the academic (STS) community has, in fact, shaped the political engagement rhetoric as well as its successor: Responsible Research and Innovation (RRI), which signals a partial departure from the idealistic notions of public participation and a renewed emphasis on innovation. Macq, Tancoigne and Strasser explain: “In other terms, the effort of policymakers to embed public participation in scientific research more broadly remained confined to the science-society programs and did not spread beyond” [8, p. 506].

## 7. Conclusion

The ‘engagement’ rhetoric is an expression of a fundamental problem that has haunted modern democratic societies for some time. As the decisions facing them have become ever more complex, governments have had to rely increasingly on the specialized advice of a scientific-technical elite of experts, resulting in a widening gap between governments and their electorates as well as an inescapable loss of legitimacy. This development has been met with attempts to re-capture citizens’ participation, be it with institutional innovations that engage ‘micro-publics’ in specialized decisions or with rhetoric calling for an unspecified engagement of the public at large. In the theoretical discussion, an attempt is made to straddle the contradiction between general democratic participation and elite expert knowledge through concepts like ‘democratization of expertise’.

In investigating the development of the rhetoric over the last two decades, we observe a progressing generality and increasing ambition. It took almost a decade and a half for the rhetoric to reach its greatest impact in the political arena, with calls to ‘engage’, science communication programs and a host of events designed to bring science to the public. Likewise, it has taken considerable time for the academic community concerned to face the inescapable paradox of the ‘engagement’ project, i.e. that it remains a top-down enterprise. This demonstrates the enormous attraction of buzzwords or rhetoric that is distant from and above a reality that they intend to describe or even create. Nevertheless, it simultaneously confirms the immovable sociological observation of the functional differentiation of modern societies and the relative stability of institutions. Rather than succeeding in its intended purpose of re-establishing legitimacy for the institution of science and democratic governments by bridging the gap between them, it seems that the ‘engagement’ rhetoric has not only outpaced practice, but also become diluted in the process.

### 7.1. Study limitation

In the current study, we analyzed only 19 policy documents, compared to a much larger set of academic articles. It should be noted that it is not possible to select policy documents with the same rigorous inclusion or exclusion criteria that can be applied to the selection of academic texts. However, the term “public engagement” spilled over into the policy arena following increased attention in the academic arena, and therefore started to appear in policy documents more recently, compared to its occurrence in academic articles. Earlier policies regarding the relationships between science and society mostly refer to concepts such as “science literacy” or “public understanding of science”. It would be highly meaningful to follow up this analysis with a study involving qualitative interviews with science policymakers in order to gain a richer understanding of their motivations, objectives and intentions with policy directives relevant to public engagement with science. However, that is unfortunately beyond the scope of the current study.

## Supporting information

S1 Appendix Codebook for “public engagement with science—origins, motives and impact in academic literature and science policy”. (DOCX)

S2 Appendix Complete list of academic articles analyzed for “public engagement with science—origins, motives and impact in academic literature and science policy”. (DOCX)

S3 Appendix Table of all policy documents analyzed for “public engagement with science—origins, motives and impact in academic literature and science policy”. (DOCX)
