# Correction: Public engagement with science—Origins, motives and impact in academic literature and science policy

**DOI:** 10.1371/journal.pone.0306474

**Published:** 2024-06-27

**Authors:** Peter Weingart, Marina Joubert, Karien Connoway

The ORCID iD is missing for the first author. Please see the author’s ORCID iD here:

• Author Peter Weingart ORCID ID is: 0000-0002-8275-525X

(https://orcid.org/0000-0002-8275-525X).

## References

[1] Weingart P, Joubert M, Connoway K (2021) Public engagement with science—Origins, motives and impact in academic literature and science policy. PLoS ONE 16(7): e0254201. 10.1371/journal.pone.0254201 34234382 PMC8263305

