# Supplementary material for: Public engagement with science—Origins, motives and impact in academic literature and science policy
Source: PLoS One. 2021 Jul 7;16(7):e0254201. doi: 10.1371/journal.pone.0254201 (PMC8263305; doi:10.1371/journal.pone.0254201)
Supplement: S1 Appendix — (DOCX) [file pone.0254201.s001.docx]

**S1 Appendix: Codebook for “public engagement with science - origins, motives and impact in academic literature and science policy”**

[V=variable]

**Exclusion criteria**

The following items were excluded from the current study, even if they contained the search terms “engage”, “engaged”, “engaging” or “engagement”:

1. Where the term(s) appeared only in the body of the article and not in the title or abstract
2. Where the term(s) appeared only in the keywords
3. Where the term(s) appeared in the title and / or abstract but wasn’t used in the context currently being studied, i.e. “engage / engagement” did not refer to the relationship between various publics and research or science communication (the science and society relationship)

**V1** Unique publication identifier (**file name**) – The full file name

**V2** **Journal name** – In which journal has the publication been published?

In the case of policy documents, this variable became **organization name,** i.e. the name of the organization that published the relevant policy document

1. JCOM – Journal of Science Communication (launched in 2002)
2. PUS – Public Understanding of Science (launched in 1992)
3. SC – Science Communication: linking theory and practice (launched in 1979)

**V3 Year of publication** – In which year was the publication published?

**V4 Title**

Copy and paste the full title of the publication (if available) or indicate “no title”

**V5 Authors (number)**

Indicate the number of authors for the publication (if available) or indicate “not provided”

In the case of policy documents, this variable was not relevant and thus left blank

**V6 Author details of first author:**

Surname; Initials
Institution (at the time of publication, affiliation of the author as provided in publication)
Country
Or indicate “not provided”

In the case of policy documents, this variable was not relevant and thus left blank

**V7 Country** (national context of the content)

Write down the main country the publication focusses on.
If equal attention is given to engagement in various countries write down up to three countries where the study was done or where the publication focusses its discussion.

If more than three countries are mentioned, indicate “multi-country”

If no specific country is evident, indicate “not evident”

**V8 Type of publication**

Type of journal publication as specified by the journal (if indicated)

In the case of policy documents, this variable was not relevant and thus left blank

1. Research article (including methods, results and discussion)
2. Other (specify; this could include comment; editorial; essay; letter; practice insight or review article [review of the state of the field / progress in an area / historical review]
3. Not clearly evident

**V9** Is “engagement” **defined** in this publication?

This refers to the use of the actual word “define” or “definition” in the context of engagement

1. Yes
2. No

**V10** If V9 = “yes”, copy the **full definition** and the relevant page number

If V9 = “no”, indicate “none”

**V11** If V9 = “no”, can any **phrase(s)** be identified that would indicate what the author(s) **consider engagement to mean**?

1. Yes
2. No

**V12** If V11 = “yes”, copy these **phrase(s)** and the relevant page number as an **informal definition**

If V11 = “no”, indicate “none”

**V13** Is “engagement” **motivated or legitimated** in this publication?

Is it defended or encouraged as a good or necessary activity?

This can be a general motivation relevant to various research fields or engagement activities. For this code, consider the question: why should we do engagement? What do the scientists hope to achieve through engagement?

1. Yes
2. No

**V14** If V13 = “yes”, choose one or more of the following terms to describe the **primary motivation**

If V13 = “no”, indicate “none”

1. Education: engaging to inform and educate the public about science, improving (general or specific) public access to scientific knowledge
2. Innovation: engaging to promote innovation, the public/citizens are considered to be a valuable source of knowledge (e.g. local expertise) and are called upon to contribute to knowledge production, bridge building and including knowledge outside ‘formal’ science
3. Legitimation: engaging to promote public trust in and acceptance of science, as well as of policies supporting science
4. Inspiration: engaging to inspire and raise interest in science, to secure a STEM educated labor force
5. Politicization: engaging to address past political injustices (historical exclusion)
6. Democratization: engaging to empower citizens to participate competently in society (democratization of society) and/or to participate in science (democratization of science)
7. Other (specify)
8. Not clearly evident

**V15** Is “engagement” **critically reviewed or discussed** in this publication?
In other words, are specific concerns, difficulties or challenges related to engagement mentioned?

1. Yes
2. No

**V16** If V15 = “yes”, choose one or more of the following **key criticisms or concerns**

If V15 = “no”, indicate “none”

1. Different socio-political/cultural environments and their influence on engagement (the dangers of generalization across countries/cultures)
2. Diversity of audiences and their needs/“single public” for engagement does not exist
3. Scientists are not prepared/trained for engagement; being in the public eye/addressing the general public runs counter to the established norms of science
4. Practical limitations of engagement, e.g. lack of time and resources, constraints of group size
5. Prior framing of engagement messages limits possibilities of broader engagement with the public
6. Tension between the audience’s/public’s expectations and scientific integrity (e.g. the audience/public calls for absolute answers while science can only provide probable ones)
7. Other (specify)
8. Not clearly evident

**V17** Is any information provided in this publication which is relevant to the **evaluation of public engagement** with science? In other words, of any evaluation formats suggested/executed?

1. Yes
2. No

**V18** If V17 = “yes”, choose one or more of the following key evaluation practices

If V17 = “no”, indicate “none”

1. Interviews
2. Formal, quantitative feedback (this includes questionnaires and surveys)
3. Informal qualitative feedback (this includes live verbal feedback at an event and written feedback like comment cards at an exhibition)
4. Other (specify)
5. Not clearly evident
