# Supplementary material for: Public engagement with science—Origins, motives and impact in academic literature and science policy
Source: PLoS One. 2021 Jul 7;16(7):e0254201. doi: 10.1371/journal.pone.0254201 (PMC8263305; doi:10.1371/journal.pone.0254201)
Supplement: S2 Appendix — (DOCX) [file pone.0254201.s002.docx]

**S2 Appendix: Complete list of academic articles analyzed** **for “public engagement with science - origins, motives and impact in academic literature and science policy”**

(N=86)

**Academic articles**

In alphabetical order according to author surname

Allspaw KM. Engaging the public in the regulation of xenotransplantation: would the Canadian model of public consultation be effective in the US? Public Understanding of Science 2004;13(4):417–28.

Amodio L. Are museums places where science and society can really engage in a dialogue? A positive example related to the rubbish emergency in the Campania region. Journal of Science Communication 2008. DOI: 10.22323/2.07010303

Árnason V. Scientific citizenship in a democratic society. Public Understanding of Science 2012;22(8) 927–40.

Barnett J, Burningham K, Walker G, Cass N. Imagined publics and engagement around renewable energy technologies in the UK. Public Understanding of Science 2012;21(1):36–50.

Bauer MW, Jensen P. The mobilization of scientists for public engagement. Public Understanding of Science 2011;20(1):3–11.

Bell L. Engaging the public in technology policy: a new role for science museums. Science Communication 2008;29(3):386–98.

Bellamy R, Lezaun J. Crafting a public for geoengineering. Public Understanding of Science 2017;26(4):402–17.

Bensaude-Vincent B. The politics of buzzwords at the interface of technoscience, market and society: the case of ‘public engagement in science.’ Public Understanding of Science 2014;23(3):238–53.

Besley JC. Public engagement and the impact of fairness perceptions on decision favorability and acceptance. Science Communication 2010;32(2):256–80.

Besley JC, Nisbet M. How scientists view the public, the media and the political process. Public Understanding of Science 2011;22(6):644–59.

Brondi S, Sarrica M. Italian parliamentary debates on energy sustainability: how argumentative ‘short-circuits’ affect public engagement. Public Understanding of Science 2016;25(6):737–53.

Buckland-Nicks A, Castleden H, Conrad C. Aligning community-based water monitoring program designs with goals for enhanced environmental management. Journal of Science Communication 2016;15(3):A01.

Bud R. Science, brands and the museum. Journal of Science Communication 2016;15(6):C03.

Burgess MM. From ‘trust us’ to participatory governance: deliberative publics and science policy. Public Understanding of Science 2014;23(1):48–52.

Campos R, Araújo M. Traditional artistic expressions in science communication in a globalized world: contributions from an exploratory project developed in northeast Brazil. Science Communication 2017;39(6):798–809.

Chilvers J. Reflexive engagement? Actors, learning, and reflexivity in public dialogue on science and technology. Science Communication 2012;35(3):283–310.

Cormick C, Nielssen O, Ashworth P, La Salle J, Saab C. What do science communicators talk about when they talk about science communications? Engaging with the engagers. Science Communication 2015;37(2):274–82.

Davies SR. Constituting public engagement: meanings and genealogies of PEST in two U.K. studies. Science Communication 2013;35(6):687–707.

Davis TH. Report: engaging the public with science as it happens. Science Communication 2004;26(1):107–13.

De Vasto D, Creighton J. Inspired by the cosmos: strategies for public engagement in nonpolicy contexts. Science Communication 2018;40(6):808–18.

Dickerson-Lange SE, Bradley Eitel K, Dorsey L, Link TE, Lundquist JD. Challenges and successes in engaging citizen scientists to observe snow cover: from public engagement to an educational collaboration. Journal of Science Communication 2016;15(1):A01.

Dijkstra, AM. Analysing Dutch science cafés to better understand the science-society relationship. Journal of Science Communication 2017;16(1):A03.

Dijkstra AM, Roefs MM, Drossaert CHC. The science-media interaction in biomedical research in the Netherlands. Opinions of scientists and journalists on the science-media relationship. Journal of Science Communication 2015;14(2):A03.

Drumm IA, Belantara A, Dorney S, Waters TP, Peris E. The Aeolus project: science outreach through art. Public Understanding of Science 2015;24(3):375–85.

Einsiedel EF, Eastlick DL. Consensus conferences as deliberative democracy: a communications perspective. Science Communication 2000;21(4):323–43.

Ellis R, Waterton C, Wynne B. Taxonomy, biodiversity and their publics in twenty-first-century DNA barcoding. Public Understanding of Science 2010;19(4):497–512.

Entradas M, Bauer MM. Mobilisation for public engagement: benchmarking the practices of research institutes. Public Understanding of Science 2017;26(7):771–88.

Felt U, Fochler M, Müller A, Strassnig M. Unruly ethics: on the difficulties of a bottom-up approach to ethics in the field of genomics. Public Understanding of Science 2009;18(3):354–71.

Fleming J, Star J. The emergence of science communication in Aotearoa New Zealand. Journal of Science Communication 2017;16(3):A02.

Gary C, Dworsky C. Children's universities — a ‘leading the way’ approach to support the engagement of higher education institutions with and for children. Journal of Science Communication 2013;12(3):C04.

Horlick-Jones T, Rowe G, Walls J. Citizen engagement processes as information systems: the role of knowledge and the concept of translation quality. Public Understanding of Science 2007;16(3):259–78.

Irwin A. Constructing the scientific citizen: science and democracy in the biosciences. Public Understanding of Science 2001;10(1):1–18.

Jensen E. Highlighting the value of impact evaluation: enhancing informal science learning and public engagement theory and practice. Journal of Science Communication 2015;14(3):Y05.

Jia H, Wang D, Miao W, Zhu H. Encountered but not engaged: examining the use of social media for science communication by Chinese scientists. Science Communication 2017;39(5):1–27.

Joubert M. Country-specific factors that compel South African scientists to engage with public audiences. Journal of Science Communication 2018;17(4):C04.

King H, Dawson E, Leyva R. Highlighting the wider relevance of science centre evaluations: a reflection on the evaluation of a physics engagement programme. Journal of Science Communication 2015;14(4):A01.

Kleinman DL, Delborne JA, Anderson AA. Engaging citizens: The high cost of citizen participation in high technology. Public Understanding of Science 2011;20(2):221–40.

Kouper I. Science blogs and public engagement with science: practices and opportunities. Journal of Science Communication 2010;9(1):A02.

Krabbenborg L, Mulder HAJ. Upstream public engagement in nanotechnology: constraints and opportunities. Science Communication 2015;37(4):452–84.

Kurath M, Gisler P. Informing, involving or engaging? Science communication, in the ages of atom-, bio- and nanotechnology. Public Understanding of Science 2009;18(5):559–73.

Lach D, Sanford S. Public understanding of science and technology embedded in complex institutional settings. Public Understanding of Science 2010;19(2):130–46

Lee NM, van Dyke MS. Set it and forget it: the one-way use of social media by government agencies communicating science. Science Communication 2015;37(4):533–41.

Longstaff H, Secko DM. Assessing the quality of a deliberative democracy mini-public event about advanced biofuel production and development in Canada. Public Understanding of Science 2016;25(2):252–61.

Maeseele PA. Science and technology in a mediatized and democratized society. Journal of Science Communication 2007. DOI: 10.22323/2.06010202

Makarovs K, Achterberg P. Science to the people: a 32-nation survey. Public Understanding of Science 2018;27(7):876–96.

Meckin R, Balmer A. Situating anticipation in everyday life: using sensory methods to explore public expectations of synthetic biology. Public Understanding of Science 2018;28(3):290–304.

Medvecky F, Macknight V. Building the economic-public relationship: learning from science communication and science studies. Journal of Science Communication 2017;16(2):A01.

Miah A. Genetics, cyberspace and bioethics: why not a public engagement with ethics? Public Understanding of Science 2005;14(4):409–21.

Miller S, Bowler S, Kanani S. RAS200—engaging citizens with astronomy across cultural divides. Journal of Science Communication 2018;17(4):C03.

Mizumachi E, Matsuda K, Kano K, Kawakami M, Kato K. Scientists’ attitudes toward a dialogue with the public: a study using "science cafes." Journal of Science Communication 2011;10(4):A02.

Moore A. Public bioethics and public engagement: the politics of "proper talk." Public Understanding of Science 2010;19(2):197–211.

Munshi D, Kurian PA, Morrison T, Morrison SL. Redesigning the architecture of policy-making: engaging with Māori on nanotechnology in New Zealand. Public Understanding of Science 2016;25(3):287–302.

Navid EL, Einsiedel EF. Synthetic biology in the science café: what have we learned about public engagement? Journal of Science Communication 2012;11(4):A02.

Neresini F, Bucchi M. Which indicators for the new public engagement activities? An exploratory study of European research institutions. Public Understanding of Science 2011;20(1):64–79.

Nyirenda D, Makawa TC, Chapita G, Mdalla C, Nkolokosa M, O’Byrne T, Heyderman R, Desmond N. Public engagement in Malawi through a health-talk radio programme 'umoyo nkukambirana': a mixed-methods evaluation. Public Understanding of Science 2018;27(2):229–42.

Ofori-Parku SS. “Whale deaths” are unnatural: a local NGO’s framing of offshore oil production risks in Ghana. Science Communication 2016;38(6):746–75.

O’Neill S, Nicholson-Cole S. “Fear won’t do it”: promoting positive engagement with climate change through visual and iconic representations. Science Communication 2009;30(3):355–79.

Pallett H. Public participation organizations and open policy: a constitutional moment for British democracy? Science Communication 2015;37(6):769–94.

Palmer SE, Schibeci RA. What conceptions of science communication are espoused by science research funding bodies? Public Understanding of Science 2014;23(5):511–27.

Petersen A, Anderson A, Allan S, Wilkinson C. Opening the black box: scientists’ views on the role of the news media in the nanotechnology debate. Public Understanding of Science 2009;18(5):512–30.

Pitrelli N. The crisis of the “public understanding of science” in Great Britain. Journal of Science Communication 2003;2(1):1–9.

Poliakoff E, Webb TL. What factors predict scientists’ intentions to participate in public engagement of science activities? Science Communication 2007;29(2):242–63.

Powell MC, Colin M. Meaningful citizen engagement in science and technology what would it really take? Science Communication 2008;30(1):126–36.

Riesch H, Potter C, Davies L. Combining citizen science and public engagement: the Open Air Laboratories Programme. Journal of Science Communication 2013;12(3):A03.

Riise J, Alfonsi L. From liquid nitrogen to public engagement and city planning: the changing role of science events. Journal of Science Communication 2014;13(4):C03.

Rogers-Hayden T, Pidgeon N. Moving engagement “upstream”? Nanotechnologies and the Royal Society and Royal Academy of Engineering’s inquiry. Public Understanding of Science 2007;16(3):345–64.

Rowe G, Horlick-Jones T, Walls J, Pidgeon NF. Difficulties in evaluating public engagement initiatives: reflections on an evaluation of the UK GM Nation? public debate about transgenic crops. Public Understanding of Science 2005;14(4):331–52.

Rowe G, Horlick-Jones T, Walls J, Poortinga W, Pidgeon NF. Analysis of a normative framework for evaluating public engagement exercises: reliability, validity and limitations. Public Understanding of Science 2008;17(4):419–41.

Rowe G, Poortinga W, Pidgeon NF. A comparison of responses to internet and postal surveys in a public engagement context. Science Communication 2006;27(3):352–75.

Saikkonen S, Valiverronen E. Framing engagement: expert-youth interaction in a PES event. Journal of Science Communication 2014;13(2):A03.

Sardo AM, Grand A. Science in culture: audiences' perspective on engaging with science at a summer festival. Science Communication 2016;38(2):251–60.

Schoerning E. A no-conflict approach to informal science education increases community science literacy and engagement. Journal of Science Communication 17(3):A05.

Selin C, Campbell Rawlings K, de Ridder-Vignone K, Sadowski J, Allende CA, Gano G, Davies SR, Guston DH. Experiments in engagement: designing public engagement with science and technology for capacity building. Public Understanding of Science 2017;26(6):634–49.

Silva J, Bultitude K. Best practice in communications training for public engagement with science, technology, engineering and mathematics. Journal of Science Communication 2009;8(2):A03.

Sleenhoff S, Cuppen E, Osseweijer P. Unravelling emotional viewpoints on a bio-based economy using Q methodology. Public Understanding of Science 2015;24(7):858–77.

Stilgoe J, Lock SJ, Wilsdon J. Why should we promote public engagement with science? Public Understanding of Science 2014;23(1):4–15.

Stodden V. Open science: policy implications for the evolving phenomenon of user-led scientific innovation. Journal of Science Communication 2010;9(1):A05.

Suldovsky B, McGreavy B, Lindenfeld L. Evaluating epistemic commitments and science communication practice in transdisciplinary research. Science Communication 2018;40(4):499–523.

Tang JJ, Maroothynaden J, Bello F, Kneebone R. Public engagement through shared immersion: participating in the processes of research. Science Communication 2012;35(5):654–66.

Tøsse SE. Aiming for social or political robustness? Media strategies among climate scientists. Science Communication 2013;35(1):32–55.

Tytler R, Duggan S, Gott R. Public participation in an environmental dispute: implications for science education. Public Understanding of Science 2001;10(4):343–64.

Von Roten FC. Gender differences in scientists’ public outreach and engagement activities. Science Communication 2011;33(1):52–75.

Ward V, Howdle P, Hamer S. You & your body: a case study of bioscience communication at the University of Leeds. Science Communication 2008;30(2):177–208.

Watermeyer R. Measuring the impact values of public engagement in medical contexts. Science Communication 2012;34(6):752–75.

Wilkinson C, Bultitude K, Dawson E. “Oh yes, robots! People like robots; the robot people should do something”: perspectives and prospects in public engagement with robotics. Science Communication 2011;33(3):367–97.

Winter E. Public communication of science and technology German and European perspectives. Science Communication 2004;25(3):288–93.
