# Supplementary material for: Public engagement with science—Origins, motives and impact in academic literature and science policy
Source: PLoS One. 2021 Jul 7;16(7):e0254201. doi: 10.1371/journal.pone.0254201 (PMC8263305; doi:10.1371/journal.pone.0254201)
Supplement: S3 Appendix — (DOCX) [file pone.0254201.s003.docx]

**S3 Appendix: Table of all policy documents analyzed for “public engagement with science - origins, motives and impact in academic literature and science policy”**

(N=19)

| **Region or country** | **Year**  **published** | **Title** | **Publisher** |
| --- | --- | --- | --- |
| United Kingdom | 2007 | Public engagement with research strategy | Research Councils UK |
|  | 2008 | A vision for science and society: a consultation on developing a new strategy for the UK | Department for Innovation, Universities & Skills |
|  | 2010 | What’s in it for me? The benefits of public engagement for researchers | Research Councils UK |
|  | 2019 | Vision for public engagement | UK Research and Innovation |
| European Union | 2013 | Public engagement in science and technology: setting the scene | European Commission. Joint Research Centre Scientific and Policy Reports |
|  | 2014 | Engage 2020. Engaging Society in Horizon 2020. Public engagement in R&I processes –  promises and demands | European Commission. Horizon 2020 Programme |
|  | 2014 | Engage 2020. Engaging Society in Horizon 2020. Current praxis of policies and activities supporting engagement in R&I – trends, needs and barriers | European Commission. Horizon 2020 Programme |
|  | 2015 | PE2020. Public Engagement Innovations for Horizon 2020. Conceptualisation of innovative public engagement | European Commission. Horizon 2020 Programme |
|  | 2015 | Engage 2020. Engaging Society in Horizon 2020. Societal engagement: policy and practice in the future | European Commission. Horizon 2020 Programme |
|  | 2015 | Engage 2020. Engaging Society in Horizon 2020. Public engagement – what’s next? | European Commission. Horizon 2020 Programme |
|  | 2016 | Citizen engagement in science and policy-making: reflections and recommendations across the European Commission | European Commission. Joint Research Centre Scientific and Policy Reports |
|  | 2016 | PE2020. Public Engagement Innovations for Horizon 2020. Boosting public and societal engagement | European Commission. Horizon 2020 Programme |
| United States of America | 2015 | Perspectives on broader impacts | National Science Foundation |
|  | 2016 | Communicating science effectively: a research agenda | National Academies of Sciences, Engineering, and Medicine |
|  | 2016 | Science literacy: concepts, contexts, and consequences | National Academies of Sciences, Engineering, and Medicine |
|  | 2018 | The current state of broader impacts: advancing science and benefiting society | National Alliance for Broader Impacts. |
| South Africa | 2015 | Science engagement strategy: science and society engaging to enrich and improve our lives | Department of Science and Technology |
|  | 2017 | Science engagement strategy implementation plan | Department of Science and Technology |
|  | 2019 | White paper on science, technology and innovation | Department of Science and Technology |
